# Small cell carcinoma arising in Barrett's esophagus: a case report and review of the literature

**DOI:** 10.1186/1752-1947-2-15

**Published:** 2008-01-22

**Authors:** Haridimos Markogiannakis, Dimitrios Theodorou, Konstantinos G Toutouzas, Andreas Larentzakis, Michael Pattas, Angeliki Bousiotou, Pavlos Papacostas, Konstantinos Filis, Stilianos Katsaragakis

**Affiliations:** 1 1st Department of Propaedeutic Surgery, Hippokrateion Hospital, Athens Medical School, University of Athens, Q. Sofias 114 av., 11527, Athens, Greece; 2 Department of Histopathology, Hippokrateion Hospital, Athens, Greece; 3 Department of Oncology, Hippokrateion Hospital, Athens, Greece

## Abstract

**Introduction:**

Gastrointestinal tract small cell carcinoma is an infrequent and aggressive neoplasm that represents 0.1–1% of gastrointestinal malignancies. Very few cases of small cell esophageal carcinoma arising in Barrett's esophagus have been reported in the literature. An extremely rare case of primary small cell carcinoma of the distal third of the esophagus arising from dysplastic Barrett's esophagus is herein presented.

**Case presentation:**

A 62-year-old man with gastroesophageal reflux history presented with epigastric pain, epigastric fullness, dysphagia, anorexia, and weight loss. Esophagogastroscopy revealed an ulceroproliferative, intraluminar mass in the distal esophagus obstructing the esophageal lumen. Biopsy showed small cell esophageal carcinoma. Contrast-enhanced chest and abdominal computed tomography demonstrated a large tumor of the distal third of the esophagus without any lymphadenopathy or distant metastasis. Preoperative chemotherapy with cisplatine and etoposide for 3 months resulted in a significant reduction of the tumor. After en block esophagectomy with two field lymph node dissection, proximal gastrectomy, and cervical esophagogastric anastomosis, the patient was discharged on the 14^th ^postoperative day. Histopathology revealed a primary small cell carcinoma of the distal third of the esophagus arising from dysplastic Barrett's esophagus. The patient received another 3 month course of postoperative chemotherapy with the same agents and remained free of disease at 12 month review.

**Conclusion:**

Although small cell esophageal carcinoma is rare and its association with dysplastic Barrett's esophagus is extremely infrequent, the high carcinogenic risk of Barrett's epithelium should be kept in mind. Prognosis is quite unfavorable; a better prognosis might be possible with early diagnosis and treatment strategies incorporating chemotherapy along with oncological radical surgery and/or radiotherapy as part of a multimodality approach. Since treatment protocols are not well established due to the rarity of the neoplasm, multi-institutional studies are needed to obtain sufficiently large populations for investigation and optimization of therapy of the disease.

## Introduction

Gastrointestinal tract small cell carcinoma is an infrequent and aggressive malignancy. It represents 0.1–1% of gastrointestinal malignancies, with the esophagus being the most common primary site [1,2]. Small cell esophageal carcinoma is a rare tumor constituting 0.8–2.4% of all esophageal carcinomas [1,2]. It is characterized by aggressive progression, high incidence of metastatic disease at presentation, and a poor overall prognosis [1,2].

Since McKeown's first description in 1952 [3], few cases of small cell esophageal carcinoma have been reported in the literature. Furthermore, only 4 cases of the neoplasm arising in Barrett's esophagus have been reported [4-7]. An extremely rare case of primary small cell carcinoma of the distal third of the esophagus arising from dysplastic Barrett's mucosa is presented and the relevant literature is reviewed.

## Case presentation

**Figure 1:**
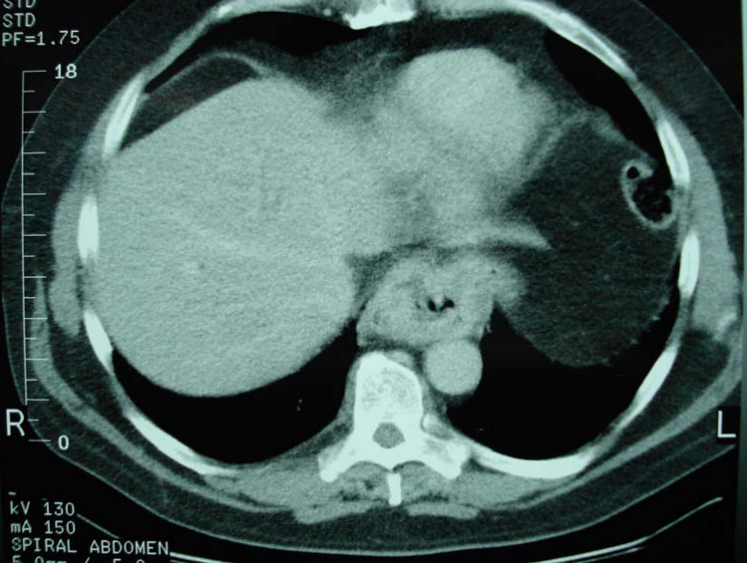
Initial CT scan revealed a large mass in the distal third of the esophagus.

A 62-year-old man with a history of gastroesophageal reflux disease presented with a one month history of epigastric pain, epigastric fullness, dysphagia, anorexia, and weight loss of 9 kgs. Clinical examination and blood tests were normal apart from elevated carcinoembryonic antigen (42.8 ng/ml; normal value < 5.0 ng/ml). Chest x-ray, electrocardiogram, echocardiography, and abdominal ultrasound (US) were normal. Esophagogastroscopy revealed an ulceroproliferative, intraluminar mass in the distal third of the esophagus obstructing the esophageal lumen. Biopsy showed small cell esophageal carcinoma. Due to esophageal lumen obstruction, endoscopic US was not feasible. Contrast-enhanced chest and abdominal computed tomography (CT) demonstrated a large tumor of the distal third of the esophagus without any lymphadenopathy or distant metastasis (Figure 1).

**Figure 2:**
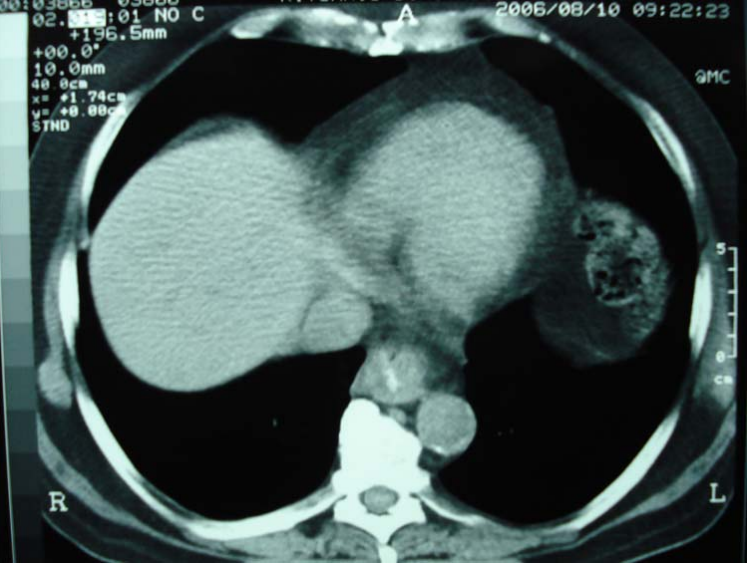
Post-chemotherapy CT scan showed a significant reduction of the tumor size.

The patient received preoperative chemotherapy with cisplatine and etoposide for 3 months with a significant reduction of the tumor size (Figure 2). En block esophagectomy with two field lymph node dissection, proximal gastrectomy, and cervical esophagogastric anastomosis were performed. After an uneventful postoperative period, he was discharged on the 14^th ^postoperative day.

**Figure 3:**
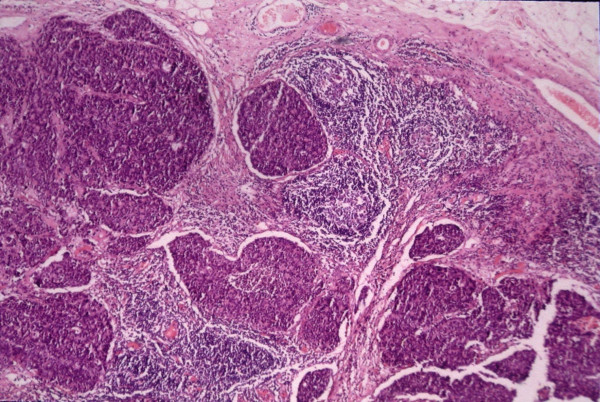
Infiltration of the esophageal wall from small cell carcinoma (H-E ×40).

Histopathology revealed a tumor (2.5 × 1.5 cm), 2 cm above the gastroesophageal junction, that was a small cell esophageal carcinoma infiltrating the submucosal and, focally, the muscular layer (Figure 3). The margins of the specimen were free of tumor. Metastasis in two paraesophageal lymph nodes was found. Moreover, Barrett's esophagus, of intestinal type, with predominantly low grade and, focally, high grade dysplasia was identified in the lower esophagus. Immunohistochemical staining of the tumor cells was positive for chromogranin and neuron-specific enolase (NSE).

A diagnosis of primary small cell carcinoma of the distal third of the esophagus arising from dysplastic Barrett's esophagus was made. The patient received another 3 month course of postoperative chemotherapy with the same agents (cisplatine and etoposide) and was free of disease at review after 12 months.

## Discussion

Small cell esophageal carcinoma is a rare neoplasm [1,2]. Although the rarity of this tumor has impeded statistical evaluation, it is generally associated with a poor prognosis because of aggressive biologic behavior and early widespread dissemination [1,2].

Small cell esophageal carcinoma is an exceptional finding in patients with Barrett's esophagus [4-7]. The presented case is indicative of the high carcinogenic risk of Barrett's epithelium. The spectrum of differentiation in Barrett's esophagus-associated carcinomas is attributed to the totipotential cell population at the squamocolumnar junction and to the considerable histologic heterogeneity of Barrett's mucosa.

The most common symptoms are dysphagia, anorexia, weight loss, chest pain, and regurgitation [1,2,6]. The neoplasm often appears as an exophytic mass usually located in the middle or lower esophagus while ulceration of the overlying mucosa is common [1,2,6]. Diagnosis is established by the characteristic silver affinity of the tumor cells, the ultrastructural occurrence of neurosecretory granules, and immunohistochemically detectable markers, including neuron-specific enolase, chromogranin, and synaptophysin [1,2,4]. Ectopic hormonal secretion, including adrenocorticotropic hormone, calcitonin, gastrin, somatostatin, and antidiuretic hormone may occur [1,2].

Although treatment protocols are not well established because of the paucity of cases and the lack of large studies, chemotherapy remains the treatment of choice given the systemic nature of the disease [1,2]. Since small cell carcinoma of the esophagus is histologically identical to small cell carcinoma of the lung and, furthermore, their aggressive behavior but also chemosensitivity are similar, the chemotherapeutic agents used for small cell esophageal carcinoma are similar to those for its lung counterpart [1,2]. Radiotherapy has been used concurrently with chemotherapy to enhance local control [1,2]. In locoregional disease, the literature suggests that treatment be initiated using chemotherapy and then, if metastatic disease is still excluded, radical surgical resection be considered as a second therapy that may have a potential impact on long-term remission and long-term survival [1,2]. Survival ranges from several weeks for untreated patients to 6–24 months for those receiving therapy [1,2].

The reported patient presented with a large tumor of the distal esophagus. Our initial treatment consisted of a 3 month course of preoperative chemotherapy with cisplatine and etoposide that resulted in a significant reduction of the neoplasm. Given the response to chemotherapy, and since no metastatic disease was identified in the post-chemotherapy investigation, radical surgical resection, including en block esophagectomy with two field lymph node dissection and proximal gastrectomy, was then performed. Based on the promising results of preoperative chemotherapy, another 3 month course of postoperative chemotherapy with the same agents was administered. Although conclusions regarding treatment of such a rare clinical entity cannot be drawn from a case report, the effects of our treatment strategy seem encouraging since our patient remained free of disease at review at 12 months.

## Conclusion

Small cell esophageal carcinoma is rare and its association with dysplastic Barrett's esophagus is extremely infrequent. The high carcinogenic risk of Barrett's epithelium, though, should be kept in mind. Prognosis is quite unfavorable and treatment protocols are not well established. A better prognosis might be possible with early diagnosis and treatment strategies incorporating chemotherapy along with oncological radical surgery and/or radiotherapy as part of a multimodality approach. Our treatment strategy of preoperative chemotherapy followed by radical surgical resection and postoperative chemotherapy in the reported patient may have yielded promising results. Multi-institutional studies are needed to obtain sufficiently large populations for investigation and optimization of therapy of the disease.

## Competing interests

The author(s) declare that they have no competing interests.

## Authors' contributions

HM contributed to manuscript conception, research, acquisition of data, drafting and writing of the manuscript. DT carried out the operation and contributed to acquisition of consent and critical review of the manuscript. KGT assisted in the operation, contributed to organising and drafting of the manuscript, and critically revised the manuscript. AL contributed to manuscript conception, research, acquisition of data, drafting and writing of the manuscript. MP contributed to manuscript conception, research, acquisition of data, drafting and writing of the manuscript. AB carried out the histopathologic evaluation and contributed to writing of the manuscript. PP contributed to the preoperative and postoperative management of the patient and to critical review of the manuscript. KF contributed to organising and drafting of the manuscript, and critically revised the manuscript. SK assisted in the operation and contributed to critical review of the manuscript.

All authors read and approved the final manuscript.

## Consent

Written informed consent was obtained from the patient for publication of this Case report and any accompanying images. A copy of the written consent is available for review by the Editor-in-Chief of this journal.
